# Development of a vivo rabbit ligated intestinal Loop Model for HCMV infection

**DOI:** 10.1186/s40104-016-0129-1

**Published:** 2016-12-13

**Authors:** Jin Tang, Qiaoxing Wu, Xinming Tang, Ruihan Shi, Jingxia Suo, Guangping Huang, Junqing An, Jingyuan Wang, Jinling Yang, Wenzhuo Hao, Ruiping She, Xun Suo

**Affiliations:** 1 National animal protozoa laboratory,College of VeterinaryMedicine, China Agricultural University, Beijing, 100193 China; 2 Laboratory of Animal Pathology & Public Health, College of Veterinary Medicine, China Agricultural University, Beijing, 100193 China

**Keywords:** HCMV, HEV, Inoculating ligated intestine *in vivo*, Immunohistochemistry and confocal immunofluorescence, Pathological lesion, Rabbit sacculus rotundus, Vermiform appendix

## Abstract

**Background:**

Human Cytomegalovirus (HCMV) infections can be found throughout the body, especially in epithelial tissue. Animal model was established by inoculation of HCMV (strain AD-169) or coinoculation with Hepatitis E virus (HEV) into the ligated sacculus rotundus and vermiform appendix in living rabbits. The specimens were collected from animals sacrificed 1 and a half hours after infection.

**Results:**

The virus was found to be capable of reproducing in these specimens through RT-PCR and Western-blot. Severe inflammation damage was found in HCMV-infected tissue. The viral protein could be detected in high amounts in the mucosal epithelium and lamina propria by immunohistochemistry and immunofluorescense. Moreover, there are strong positive signals in lymphocytes, macrophages, and lymphoid follicles. Quantitative statistics indicate that lymphocytes among epithlium cells increased significantly in viral infection groups.

**Conclusions:**

The results showed that HCMV or HEV + HCMV can efficiently infect in rabbits by vivo ligated intestine loop inoculation. The present study successfully developed an infective model *in vivo* rabbit ligated intestinal Loop for HCMV pathogenesis study. This rabbit model can be helpful for understanding modulation of the gut immune system with HCMV infection.

## Background

Human cytomegalovirus (HCMV) is one of important viruses that cause congenital infection in humans worldwide. HCMV is also the leading viral cause of birth defects associated with infections and a major cause of morbidity and mortality in transplant patients.

Human cytomegalovirus (HCMV) belongs to the Beta-herpesvirinae subfamily, which also includes cytomegaloviruses from other mammals; humans and monkeys serve as natural hosts. There are currently eight species in the CMV genus, but rabbit CMV is not included. HCMV is found ubiquitously and infects as many as 80% of healthy adults [1]. HCMV likes to infect epithelial tissue and causes hepatitis, pneumonitis, and chronic gastroenteritis. HCMV can vertically transmitte and causes congenital abnormalities in humans all around the world [2]. Cell-mediated immunity, including cytotoxic T lymphocytes (CTLs) and nature Killer (NK) cells, is essential for limiting and clearing viral infections [3, 4]. UL16 protein encoded by HCMV can suppress the ligands ULBP1, ULBP2 and MICB of NK cell receptors and results in increased viral load during immune evasion [5].

Cytomegaloviruses are highly species-specific . However, mouse CMV (MCMV) and human CMV (HCMV) have cross-infections among species [6]. The inhibition of cross-species infections are likely due to no interactions between virus and host protein. [7]. The mechanism of HCMV infections has been studied by cell culture and using animal models that CMV infects their own hosts.

In immune competent animals, CMV usually causes asymptomatic latent infection with low levels of viral shedding. However, in immunocompromised individuals such as HIV-infected persons, organ transplant recipients, and developing fetuses, HCMV can cause severe diseases and even become life-threatening with high levels in many organs [8]. In preliminary test of our Lab, previous data demonstrated that HEV could be detected at 10 min post-infection, Therefore, HCMV-HEV co-infection group in the present study could be considered as immunocompromised modles.

CMV pp65 gene encode matrix phosphoprotein targete nucleus immediately after infection. [9]. Clinical diagnosis is based upon the detection of pp65 by CMV antigenemia test. [10]. CMV UL16 gene encoded glycoprotein is synthesized soon after virus penetration and accumulates in both nucleus and cytoplasm [11]. These two early HCMV genes are expressed in the nucleus of infected cells within 1 to 24 h of infection, prior to viral DNA replication. During infection, pp65 is a major target of humoral and cellular (CD4 and CD8 T-cell) immune responses [12]. There was evidence that pp65 modulates antigen presentation [13]. Many other viral gene products encoded during replication have been shown to modulate viral and host cell processes [14–17].

Ligated intestine *in vivo* animal models have been used for studying mechanisms of intestinal infection and enterotoxin to reveal the mutual effects between pathogens and intestinal mucosal epithelium [18–22]. Intestinal tract is one of the most common sites for HCMV infection [23]. The sacculus rotundus and appendix are peripheral immune organsin intestinal tract, which are ideal sites for study immune activities of HCMV.

In this study, we observed pathological changes of ligated sacculus rotundus and vermiform appendix inoculated with HCMV and strain of rabbit HEV in rabbit. Our results indicate that HCMV or HEV + HCMV can efficiently infect in rabbits by vivo ligated intestine loop inoculation. We successfully developed an infective model *in vivo* rabbit ligated intestinal Loop for understanding modulation of the gut immune system with HCMV infection, as well as other viruses infection.

## Methods

### Ethics statement

All animal experiments involved in this study were approved by the Animal Care and Use Committee of China Agricultural University (CAU) . (Permit Number: 20150512–212). We followed guidelines of CAU Animal Care and Use Committee for experimental animals during this study.

### Experimental design and procedures for inoculation

Nine rabbits were randomly divided into three groups. Each rabbit ligated intestine was inoculated with viruses or PBS in the sacculus rotundus and vermiform appendix. Group 1 was only inoculated with 0.5 mL (50.00 TCID50) HCMV (single infection group, SI). Group 2 was inoculated with 0.5 mL (50.00 TCID50) HCMV and 0.5 mL (2.746 × 10^7^ genome equivalents per mL) HEV (double infection/co-infection group, DI). Group 3 was injected with PBS (control group, CT). Tissue from sacculus rotundus, vermiform appendix and distal appendix were collected 1 and 0.5 h post-infection and stored at −80 °C for further use.

### Virus

The strain of rabbit HEV was derived from the second passage of a fecal sample from a rabbit infected with rabbit HEV (rhBJ1, accession number KF648530). A 10% suspension of positive feces was prepared and titered with RT-PCR, as described previously [24]. The titer of the suspension was 2.746 × 10^7^ genome equivalents per mL. The viral suspension was stored at −86 °C for later use.

HCMV AD169 strain was obtained from the CDC China. CMV AD169 was propagated in human fibroblasts cell line MRC-5. The virus had been passed many generations in the cells to increase the CPE to 50.0 log TCID 50/mL Virus titer was determined in the MRC-5 cells according to Reed-Muench method. Virus supernates were purified by centrifugation at 1,000 × g for 10 min at 4 °C and stored at −80 °C until use.

### Animal

Eighty-day-old male rabbits were purchased from Xing Long Experimental Animal Center, Beijing, China. Before inoculation, the sera of the rabbits were confirmed to be negative for HEV and HCMV antibodies with reverse transcription-nested PCR (RT-nPCR).

### Sampling

The samples were obtained from rabbits sacrificed 1.5-h post-infection. Inoculated sacculus rotundus, vermiform appendix, and uninoculated distal appendix were collected. These samples were divided into two parts. One part was fixed in paraformaldehyde-glutaraldehyde buffer for histopathology observation. Another part was washed with PBS 3 times to rinse out intestinal contents, and stored in −80 °C for further use.

Other than specifications, the followings are the abbreviations of collected tissues:SISR (single infection sacculus rotundus), DISR (double infection sacculus rotundus), CTSR (control sacculus rotundus); SIVA (single infection vermiform appendix), DIVA (double infection vermiform appendix), CTVA (control vermiform appendix); SIDA (single infection distal appendix), DIDA (double infection distal appendix), CTDA (control distal appendix).

### Antibody

The rabbit anti HCMV pp65 antibody was purchased from Bio-Science Company. The mouse anti rabbit HEV ORF2 antibody was purchase from Beijing Protein Institute. Second fluorescence antibodies were purchased from Bio-Science company.

### RT-PCR

Samples were washed with PBS trice and grinded in liquid nitrogen. UL16 and pp65 DNA of HCMV detected by PCR. Total DNA was extracted from intestinal samples using a Universal Gen DNA kit(CWBIO, Beijing, China). The primers for Immediate-Early gene pp65 of HCMV were (5′-CACCTGTCACCGCTGCTATATTTGC – 3′, 5′-CACCACGCAGCGGCCCTTGATGTTT -3′). The primers for Early gene UL16 were (5′-CGCGGTACCATGGAGCGTCGCCGAGGTACTGTAC-3′, 5-TGGAACATCGAATGGGTAGTCCTCGGTGCGTAA-3′).

The HEV conservative sequence was tested by nested RT-PCR [24]. Total RNA was isolated in Trizol reagent. The outer primer pairs were 5′-AATTATGCYCAGTAYCGRGTTG-3′ and 5′-CCCTTRTCYTGCTGMGCATTCTC-3′; and The inner primer pairs were 5′-GTWATGCTYTGCATWCATGGCT-3′ and 5′-AGCCGACGAAATCAATTCTGTC-3′.

### Western-blot

Tissues were grinded in liquid nitrogen and lysed in RIPA buffer with cocktail of protease inhibitor. The lysates were loaded into 12% SDS-PAGE gel under reducing condition. Proteins were electrotransferred onto a nitrocellulose membrane. The membranes were blocked, probed separately with affinity-purified pp65 Ab or anti-ORF2-specific Ab for 1 h, followed by incubation with HRP-conjugated rabbit anti-mouse or donkey anti-rabbit antibody. All blocking, incubation, and washing were performed in 5% non-fat milk and 0.05% Tween 20 in PBS. Proteins were visualized by the enhanced chemiluminescence detection (Pierce) method according to the instructions of the manufacturer. For reprobing, the blots were first incubated in stripping buffer (Fisher) for 15 min at room temperature and then washed fully to remove residual reducing agent.

### Histopathological examination

The sacculus rotundus and vermiform appendix were collected and fixed in 2.5% glutaraldehyde– polyoxymethylene solution for 48 h. The fixed tissues were routinely processed, embedded in paraffin, sectioned (4 μm thickness), Each paraffin block was cut and spreaded 5 slices, separately stained with hematoxylin and eosin(HE) for light microscopic observation, immunohistochemistry and confocal immunofluorescence.

### Immunohistochemistry and confocal immunofluorescence

Immunohistochemical staining was performed according to the instructions of the Histostain™-Plus Kit (ZSGB-BIO, Beijing, China). Briefly, paraffin wax sections were deparaffinized and rehydrated. Sections were pretreated with 3% H_2_O_2_ for 10 min, and then the sections were blocked with blocking buffer containing 3% normal goat serum (NGS) for 30 min. Monoclonal mouse anti-HEV ORF2 antibody (1:300 dilution) or rabbit anti-HCMV pp65 antibody was used as the primary antibody and incubated at 37 °C for 2 h. followed by treatment with streptavidin-peroxidase for 30 min at 37 °C. Color was developed with 3,3′- Diaminobenzidine tetrahydrochloride (DAB; ZSGB-BIO, Beijing, China) for 5 min. Gill’s hematoxylin was then applied for counter stain. The primary antibody was replaced with phosphate-buffered saline in the negative control. The slides were then observed under an Olympus microscope (Japan).

For confocal immunofluorescence, after incubation of the 1st antibody, the slices were then incubated with FITC-conjugated goat anti-rabbit IgG and Cy3-conjugated goat anti-mouse IgG in blocking buffer. After each step, slides were washed at least three times with 0.1% Tween 20 in PBS. Coverslips were mounted on slides with ProLong antifade kit (Molecular Probes) and examined using a Leica LSM 510 confocal fluorescence microscopy. The images were processed using the Image Examiner software (Leica).

### Quantitative statistics method for comparing lymphocytes and goblet cells in epithelial tissue

Under 40 times the objective, observed tissue slice, calculate the intraepithelial lymphocytes(IELs) and intraepithelial goblet cells(IGCs) among 100 mucosal epithelium cells of the villus tip and the two sides of dome. There were 8 microscopic views to be calculated for each tissue. The GraphPad Prism software was used for statistical analysis. The parameters were analyzed as means and standard deviation. Differences were considered to be statistically significant with *P* < 0.05 indicated as one asterisk and *P* < 0.01 as double asterisks.

## Results

### RT-PCR detection of HCMV and HEV RNA in the ligated SR and VA of living rabbits

**Fig. 1:**
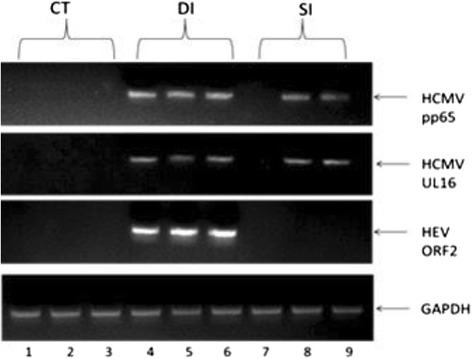
PCR detection for HCMV PP65 DNA and HEV ORF2 RNA. Lanes 1\2\3 are samples from control group. Lane 1 was CTDA, Lane 2 was CTSR, Lane 3 was CTVA. We were unable to amplify the DNA of the HCMV pp65and UL16, and RNA of HEV ORF2. Lanes 4\5\6 were samples from double infection group. Lane 4 was DIDA, Lane 5 was DISR, Lane 6 was DIVA. We were successfully amplified all the genes of each lane, especially lane 4 which was from non-injected distal bowel, still showed positive for the injected virus. This indicated that these viruses penetrated into tissue rapidly. Lanes 7\8\9 were samples from single infection (HCMV alone) group. Lane 7 was SIDA, Lane 8 was SISR, Lane 9 was SIVA. We did not detect any signalof HEV RNA. We detected HCMV RNA in the SR and VA but not in DB. GAPDH was an internal loading control

HCMV pp65 is the early-late lower matrix phosphoprotein, a major constituent of CMV virion. CMV pp65 is abundantly synthesized during lytic infection. CMV UL16 is a glycoprotein synthesized soon after infection. These two genes are expressed in 1–24 h post-infection. We extracted RNA from CT, SI and DI group respectively and amplified with different primers. To determine whether viral infection succeeded, we used both tissue inoculated with virus and without for the RT-PCR test. The vermiform appendix, sacculus rotundus, and distal appendix RNA samples were run, respectively, in each group. The results of detection of HCMV and HEV RNA in the ligated SR and VA of living rabbits showed as following Fig. 1. The samples from rabbits in the control group (CT) were negative for HCMV DNA and HEV RNA. HCMV DNA detection was performed with the amplification of a 400 bp DNA product of the tegument protein pp65 and a 500 bp DNA product of glycoprotein UL16 from both SI and DI group. HEV RNA was detected by conventional nested RT-PCR, which is positive only in the DI group.

### HCMV encoded protein pp65 expressed in co-infection group

**Fig. 2:**
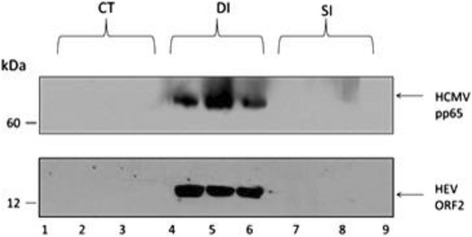
HCMV pp65 and HEV ORF2 protein expression in infected tissues. Lanes 1\2\3 were samples from the control group. Lane 1 was CTDA, Lane 2 was CTSR, Lane 3 was CTVA. We were unable to detect any protein for the HCMV pp65 or HEV ORF2. Lanes 4\5\6 were samples from the double infection group. Lane 4 was DIDA, Lane 5 was DISR, Lane 6 was DIVA. We successfully detected HCMV pp65 and HEV ORF2 in each lane. Particularly, lane 4, which was uninjected distal appendix, still shows positive for the injected viral protein. This indicates that these viruses penetrated into tissue and synthesized early protein rapidly. Lanes 7\8\9 were samples from the single infection (HCMV alone) group. Lane 7 was SIDA, Lane 8 was SISR, Lane 9 was SIVA. We did not detect any signal in the SI group

The results of HCMV encoded protein pp65 expressed in co-infection group showed as following Fig. 2. There were no bands from the CT group. For the SI group, there were barely visible bands from the vermiform appendix and sacculus rotundus. However, we can detect clearly 65kDa bands were detected in the DI group, which represented the expression of pp65 protein. HCMV pp65 antibody was stripped down after chemiluminescene detection and the membrane was reprobed with anti-HEV ORF2 antibody. Both CT and SI groups were HEV ORF2 negative. However, HEV ORF2 was detected in DI group, which was consistent with the RT-PCR results.

### Histopathology observation

#### Ligated sacculus rotundus

**Fig. 3:**
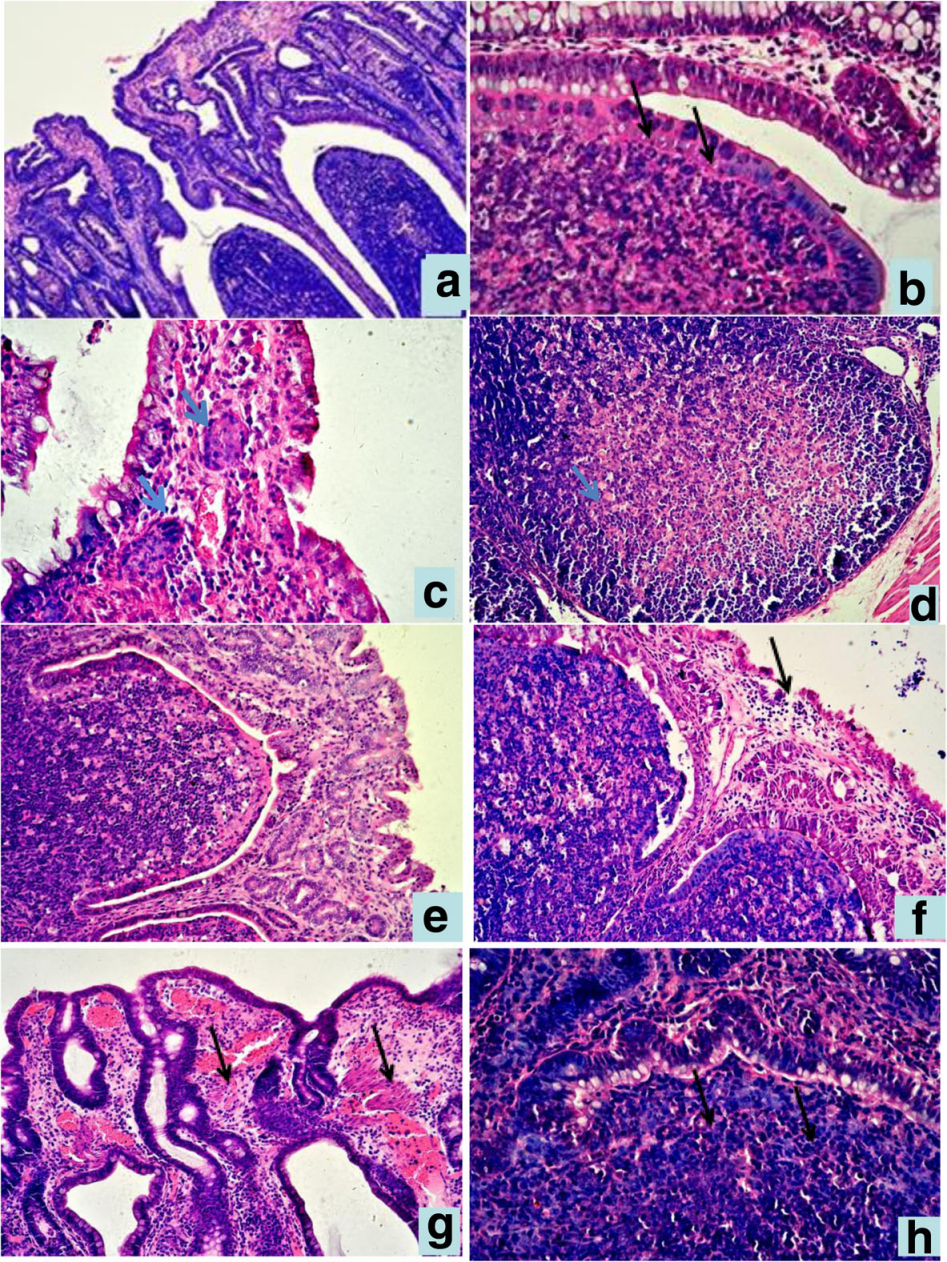
Histopathological lesions of SR and VA in different groups,HE staining. **a** SR in CT group. 10 × ; **b** Lymphoid follicle dome of SR in SI group.40 × ; **c** epithelium villus tip of SR in DI group. There were two symplasms in lamina propria(↑). 40 × ; **d** Lymphocyte depeletion and necrosis of lymphoid follicle of SR in DI group. 20 × ; **e** Epithelium and Peyer’s pathches of VA in CT group. 20 × ; **f** Mucosal epithelium of VA was degenerate, exfoliated in SI group,20 × ; **g** Hyperemia, hemorrhage in lamina propria of VA in DI group, 20 × ; **h** the boundary between epithelium and lymphoid tissue unclear. in dome of VA in DI group, 40×

In the CT group, epithelial cells are intact with normal structures of lamina propria and Peyer’s patches. The dome epithelium is clearly visible. The boundary between epithelial and lymphoid tissue is clear (Fig. 3a). In the SI group, epithelium in the villus tip of SR was slightly degenerated with edematous lamina propria. Capillaries were congested. The layer of mucosal epithelium had more l ymphocytes. Moreover, lymphocytes in the lymphoid follicle dome of Payer’s patches in the submucosa were slightly evacuated. The number of lymphocytes in the dome was increased markedly, but the boundary between epithelium and lymphoidtissue was clear (Fig. 3b). Lymphocytes in the germinal center of the follicle were slightly evacuated.

In the DI group, histological lesions are more severe than SI group. Epithelium in the villus tip of SR are distinctly degenerated and exfoliated with distinct edema of the lamina propria. Capillaries were distinctly enlarged and congested. Multifocal hemorrhages were noted. There were intranuclear inclusion bodies formed in epithelial cell and lymphocyte infected with the virus, In one SR mucosal lamina propria, several symplasms were found (Fig. 3c). Moreover, lymphocytes in the lymphoid follicle dome of Payer’s patches in submucosa were distinctly evacuated. Lymphocytes in the dome increased largely, making the boundary between epithelium and lymphoid tissue unclear. Lymphocytes migrated in dome; lymphocytes in germinal center of follicle were evacuated significantly and even showed necrosis (Fig. 3d). Capillaries in the submucosa were distinctly enlarged and congested.

#### Ligated vermiform appendix

Epithelium of the appendix is thinner than that of SR. In the CT group, the structure of epithelium is intact with normal lamina propria and enteraden (Fig. 3e). In the SI group, epithelium in the villus tip of the VA was slightly degenerate, exfoliated, and hyperplastic. There was slightly edema of the lamina propria, with a large number of lymphocytes. The number of lymphocytes among the epithelium is significantly increased. Lymphocytes in Payer’s patches of the submucosa evacuated inordinately, lymphocytes in the dome epithelium increased largely, but the boundary between epithelial and lymphoid tissue is clear. Lymphocytes in the germinal center of lymphoid follicle evacuated slightly (Fig. 3f).

In DI group, similer, but more severe histology lesions were observed in this group. Epithelium in the villus tip of SR are distinctly degenerate, exfoliated, and hyperplastic. There was distinct edema of the lamina propria. Capillaries were distinctly enlarged and congested. There were multifocal Interspersed hemorrhage (Fig. 3g). Intraepithelial lymphocytes of the dome increased largely in number to make the boundary between epithelium and lymphoid tissue unclear (Fig. 3h).

### Immunohistochemistry detection of HCMV pp65 and HEV ORF2 antigens in ligated SR and VA

#### HCMVpp65 antigen distribution and location in ligated SR

**Fig. 4:**
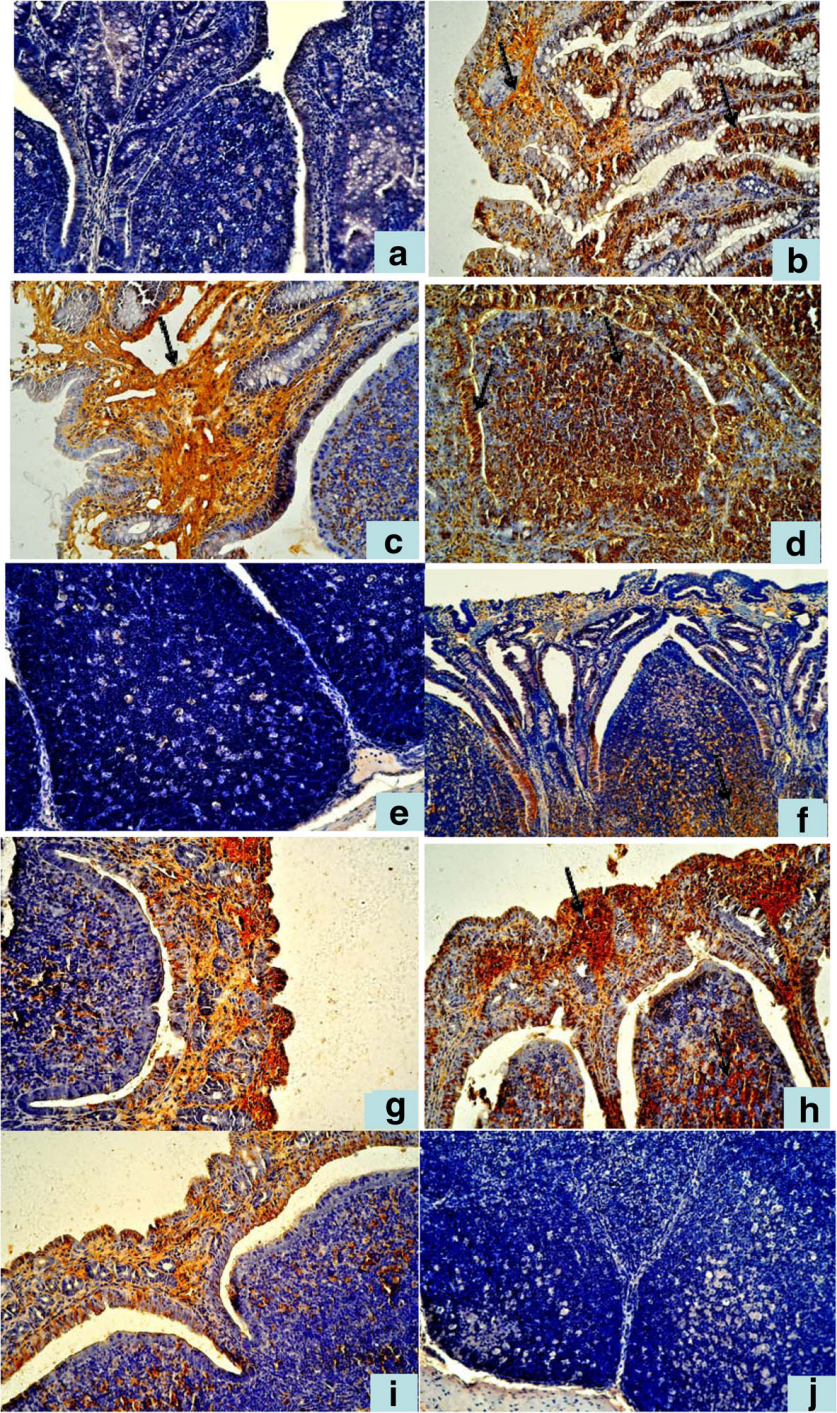
Immunohistochemistry detection of HCMV pp65 and HEV ORF2 antigens in ligated SR and VA. **a** No positive HCMV pp65 signal in the SR in CT group. 20 × ; **b** Positive HCMV pp65 signal in the mucosal epithelial of SR in SI group. 20 × ; **c**, **d** In the DI group,stronger positive HCMV pp65 signal than in the SI group. 20 × ; **e** No positive HEV ORF2 signal in the Peyer’s patches of SR in CT group. 20 × ; **f** Positive HEV ORF2 signal in SR in DI group.20 × ; **g** Positive HCMV pp65 signal in the mucosal epithelial and dome of VA in SI group 20 × ; **h** In the DI group,stronger positive HCMV pp65 signal than in the SI group. 20 × ; **i** Positive HEV ORF2 signal in the mucosal epithelium and dome of VA in DI group. 20 × ; **j** No positive HEV ORF2 signal in the lymphoid follicles of VA in CT group. 20×

In the CT group, no distinct positive signals can seen in the epithelium and Peyer’s patches (Fig. 4a). In the SI group, each tissue showed strong HCMV positive antigenic response. There were distinctly positive signals (showed in brown) in the mucosal, glandular, and dome epithelium, especially in the nucleus and cytoplasm of the lamina propria (Fig. 4b). Nucleus and cytoplasm of lymphocytes and macrophages in the dome and follicle germinal center show an inordinately positive signal. In the DI group, we observed similar, but stronger positive than in the SI group (Fig. 4c, d).

#### HEV ORF2 antigen distribution and location in ligated SR

CT group (Fig. 4e) was HEV ORF2 negative. HEV ORF2 antigenic positive signal was only observed in the DI group, but not in the SI group. There were distinctly positive signals in the mucosal epithelium, glandular epithelium, and cytoplasm of the dome epithelium. Lymphocytes and macrophages in dome and follicle germinal center showed an inordinately positive signal (Fig. 4f).

#### HCMVpp65 antigen distribution and location in ligated VA

The observation of HCMV pp65 antigen distribution in ligated VA was consistence with SR. There were distinctly positive signals in the mucosal, glandular, and dome epithelium, especially in the nucleus and cytoplasm of the lamina propria in both SI (Fig. 4g).and DI groups, but the signals are much stronger in the DI group (Fig. 4h).

#### HEV ORF2 antigen distribution and location in ligated VA

The observation of HEV ORF2 antigen distribution and location in ligated VA is consistence as there was in SR. HEV ORF2 antigenic positive signal was only observed in the DI group, but not in the SI group. There are distinctly positive signals in the mucosal epithelium, glandular epithelium, and cytoplasm of the dome epithelium (Fig. 4i). In CT group no positive signals could be found (Fig. 4j).

### Immunofluorescence of HCMV pp65 and HEV ORF2 under confocal microscope

#### Colocalization of HCMV pp65 and HEV ORF2 of ligated SR

**Fig. 5:**
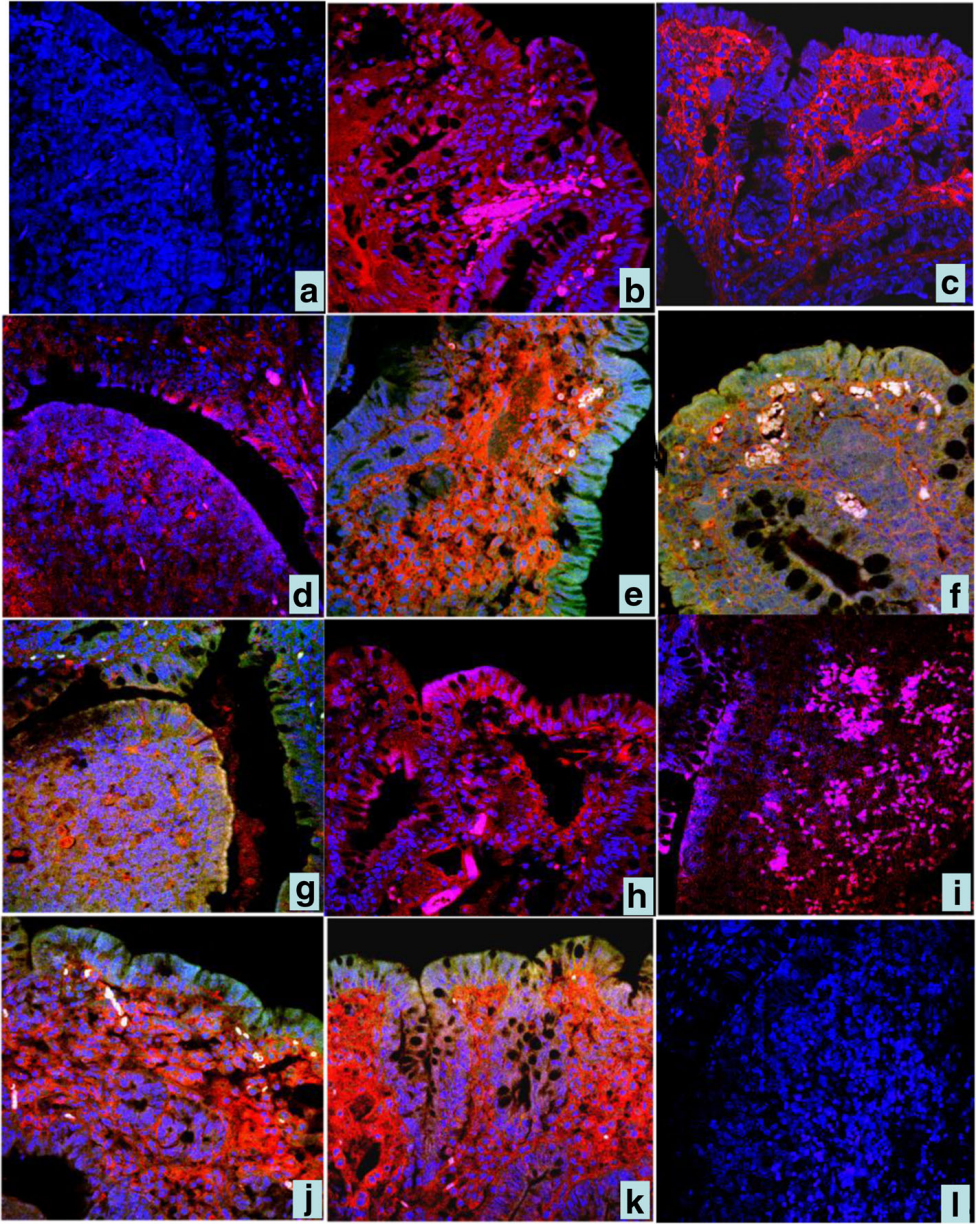
Immunofluorescence colocalization of HCMV pp65 and HEV ORF2 antigens in ligated SR and VA. **a** In the CT group, no positive signals,only the blue color DAPI were seen in the epithelium and dome. 40 ×; **b**, **c, d** In SI group, red color represents HCMV pp65 positive signal in the mucosal epithelium and dome of SR. 40 × ; **e**, **f**, **g** In DI group. green color represents HEV ORF2 positive signa, red color HCMV pp65 positive signal and yellow color represents overlapped of green and red color.in the mucosal epithelial and dome of SR. 40 × ; **h**, **i** In VA.of the SI group, red and pink color HCMV pp65 positive signal in the mucosal epithelium and dome of VA. 40 × ; **j**, **k** In DI group. green color HEV ORF2 positive signal, red color HCMV pp65 positive signal and yellow color represented.in the mucosal epithelial and dome. 40 × ; **l** In VA.of the CT group, no HCMV or HEV ORF2 fluorescence signals in the mucosal epithelial and dome . 40×

The results were consistent with immunohistochemistry. In the CT group, no distinct positive signals were seen in the epithelium and Peyer’s patches, only the blue color DAPI was seen. (Fig. 5a). In the SI group, Cy3 red staining of HCMV pp65 protein was seen. There were positive signals in the lamina propria, glandular epithelium, dome epithelium and lymphoid tissue in deep mucosa (Fig. 5b, c and d). In DI group, not only Cy3 signal but also FITC green staining representing HEV ORF2 were seen. It was common to see colocalization of red and green colors merged into yellow. In some areas of the lamina propria, and dome area, the two colors completely overlapped (Fig. 5e, f and g).

#### Colocalization of HCMV pp65 and HEV ORF2 in ligated VA

The results were similar to the SR. In the SI group, Only Cy3 red color staining of HCMV pp65 protein was seen in the lamina propria, glandular epithelium, dome epithelium, and lymphoid tissue in deep mucosa (Fig. 5h, i). In the DI group, not only Cy3 signal but also FITC green color staining, representing HEV ORF2, could be seen (Fig. 5j, k). In the CT group, no HCMV or HEV ORF2 fluorescence signals could be observed (Fig. 5l).

### Quantitative statistics for comparing IELs and IGCs in mucosal epithelium

#### Statistic data of IELs in the mucosal epithelium of the villus tip increased post-infection

**Fig. 6:**
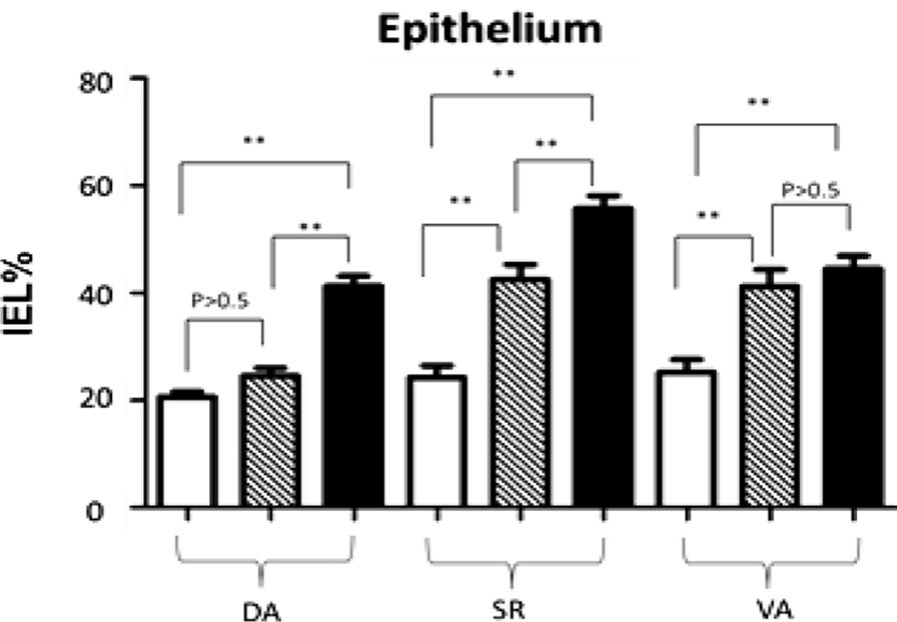
Statistic data of IELs percentage in the mucosal epithelium of villus tip post-infection In Fig. 6, blank bars represent control group CT, striped bars represent HCMV single infection group SI, and black bars represents HCMV + HEV double infection group DI

Statistic data of IELs percentage in mucosal epithelium of the villus tip post-infection was as following Fig. 6. For the uninjected distal bowel DB, there was no difference between CTDA and SIDA. However, differences of DIDA vs. CTDA and DIDA vs. SIDA were significant. For the injected sacculus rotundus, differences among each group were significant. For the injected vermiform appendix, there was no difference between CTDA and SIDA, but differences of CTVA vs. SIVA and CTVA vs. DIVA were significant.

#### IELs in the mucosal epithelium of the two sides of dome increased post-infection

**Fig. 7:**
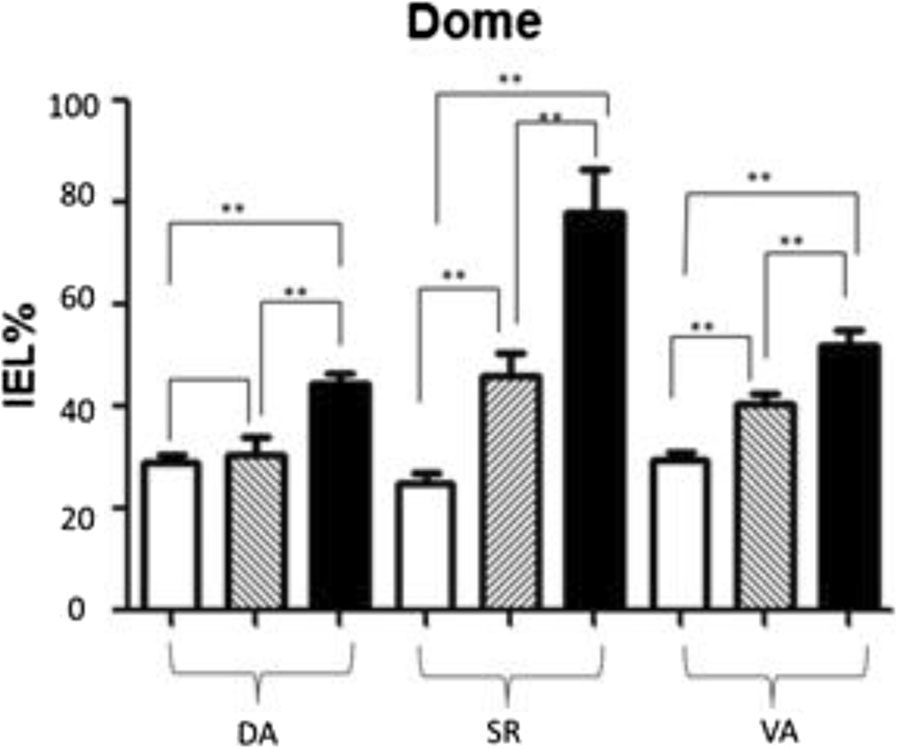
Statistic data of IELs percentage in the mucosal epithelium of two sides of dome post-infection. In Fig. 7, *blank* bars represent control group CT, *striped* bars represent HCMV single infection group SI, and *black* bars represents HCMV + HEV double infection group DI

Statistic data of IELs percentage in mucosal epithelium of two sides of dome post-infection is as following Fig. 7. The results are similar to Fig. 6. There are significant differences in each tissue of CT, SI and DI groups.

#### Statistic data of IGCs percentage in the mucosal epithelium of villus tip post-infection

**Fig. 8:**
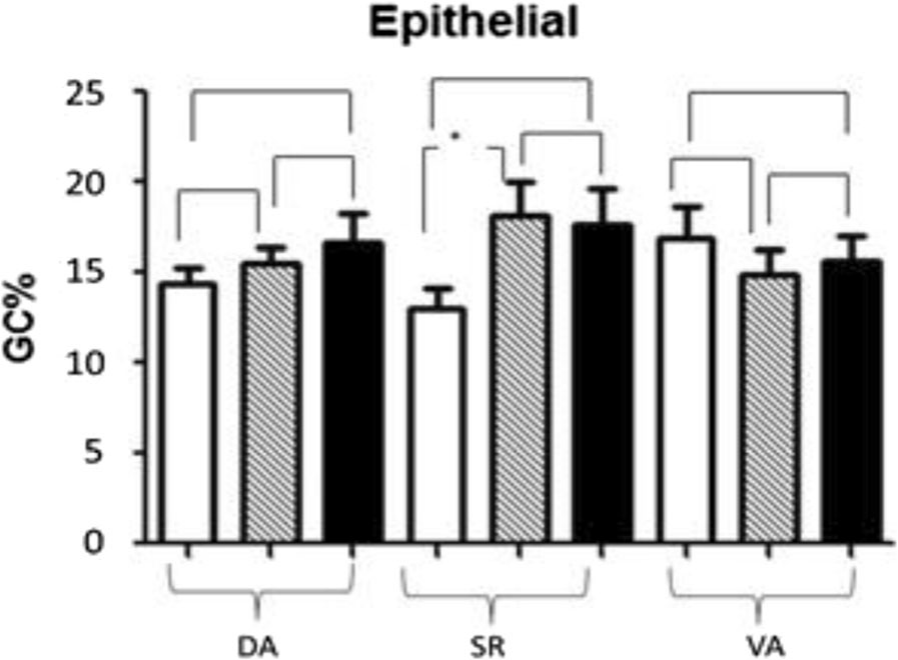
Statistic data of IGCs percentage in the mucosal epithelium of villus tip post-infection. In Fig. 8, *blank* bars represent control group CT, *striped* bars represent HCMV single infection group SI, and *black* bars represents HCMV + HEV double infection group DI

No variation of IGCs percentage in the mucosal epithelium of the villus tip post-infection (Fig. 8).

There was a difference between CTSR and SISR, but there were no differences between any other groups.

#### Statistic data of IGCs percentage in the mucosal epithelium of two sides of dome post-infection

**Fig. 9:**
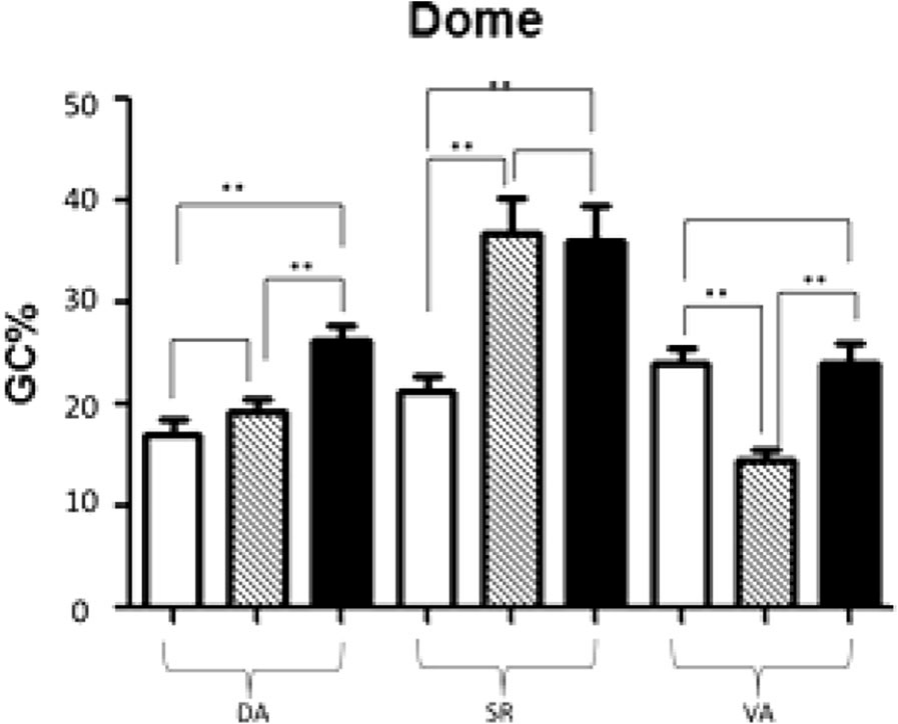
Statistic data of IGCs percentage in the mucosal epithelium of two sides of dome post-infection. In Fig. 9, *blank* bars represent control group CT, *striped* bars represent HCMV single infection group SI, and *black* bars represents HCMV + HEV double infection group DI

Statistic data of IGCs percentage in mucosal epithelium of two sides of dome post-infection was as following Fig. 9. For the uninoculated distal appendix DA, there was no difference between CTDA and SIDA. However, differences of DIDA vs. CTDA and DIDA vs. SIDA were significant.

For the injected sacculus rotundus, differences in CTSR vs. SISR and CTSR vs. DISR were significant, while there was no difference between SISR and DISR.

For the injected vermiform appendix, the lowest goblet cell percentage was SIVA, which showed significant difference with CTVA and DIVA.

## Discussion

HCMV infecting immunocompromised individuals can cause increased morbidity and mortality. The importance of congenital CMV infection as a significant public health issue is well recognized and the development of a successful vaccine is a priority. But there is still lack of animal models for the pathogenesis study of human virus because of high species sensitivity. Here, we successfully developed a vivo rabbit ligated intestinal Loop model for understanding modulation of the gut immune system with HCMV infection, as well as other viruses infection.

CMV pp65 antigenemia test utilizes an indirect immunofluorescence technique for identifying the pp65 protein of cytomegalovirus in peripheral blood leukocytes, which has proven efficacy in the detection and monitoring of immune response of CMV infection in immunocompromised patients. The advantage of this assay is the rapidity in providing results a few hours following infection. During HCMV infection, pp65 is a major antigen for cellular immune responses. CMV pp65 is also a tegument protein and involved in manipulation of the host’s immune system. HEV has three open reading frames (ORFs) of which ORF2 encodes the capsid protein. The ORF2 protein contains RNA binding activity, specifically binding the 5′ end of the HEV genome. ORF2 is synthesized as a large glycoprotein precursor that is cleaved into the mature viral capsid protein, which harbors immunoreactive epitopes; therefore, it has largely been used in diagnostics and vaccine development. In present study, we also used indirect immunofluorescence staining with tissue paraffin sections rather than peripheral blood leukocytes. The immunofluorescence signal distribution of the injected tissue paraffin sections showed the protein localization in the cells, which was more intuitive than counting the positive number of suspension cells. We used both anti-pp65 and anti-ORF2 antibodies to perform duo-tone staining. The cy3 red color represents pp65 expression, FITC green color represents ORF2 expression, and DAPI blue represents the nucleus. There is yellow color when the red and green colors merged, and white when red, green, and blue colors merged.

As a lower matrix phosphoprotein and also the main content of virion body, CMV pp65 protein is a frequent target for humoral as well as exceptionally strong CMV-specific CD8+ T cell response during lytic infection [12]. It has been common to use pp65 as an indicator for diagnosis of HCMV infection in immunocompromised individuals [10]. The significant findings of HCMV pp65 RNA and protein expression in the sacculus rotundus in this study indicate that HCMV can stimulate both immune and cellular response in the rabbit intestinal tract. which could explain why we detected pp65 protein in the DI. This could be because HCMV on its own does not activate immune response in healthy individuals.

The intestinal tract is the most common site of HCMV infection [23]. The sacculus rotundus is a peripheral immune organ containing lymphoid tissue that functions similarly to human gastro-intestinal lymphoid tissue (GALT), which is in charge of alimentary tract mucosal immunity and anti-infection [25, 26]. The dome epithelium is the tunnel for turnover of inflammatory cells, and also the barrier to block pessimum antigens [27]. The appendix is a dead-end pouch found extending alongside the margin of the ascending colon near the cecum which is in charge of sampling antigens from the contents of the intestine and determining which antigens are pathogens that require an immune response. It is important to understand the immune response here during virus infection [28]. There are four layers of sacculus rotundus and appendix: mucosa (includes epithelium, lamina propria, and muscularis mucosa), submucosa, muscular, and serosa. Highly developed lymphoid tissue in the submucosa is formed by the dome, lymphoid follicle, interfollicular area, and cap area [29, 30]. The dome epithelium is composed of cuboidal absorptive epithelial cells covering follicles which are interrupted by delicate membraneous cells. Peyer’s patches (PP) are aggregated lymphoid nodules alongside intestine mucosa which are smooth without villus and enteraden and always bulged like a dome. PP are covered by follicular associated epithelium (FAE) or dome epithelium (DE). In Peyer’s patches, antigen is transported and presented in the DE rather than in afferent lymphatic vessels in peripheral lymph nodes. Antigen from the lumen can be processed and transferred to the domes of PP through the DE [31, 32].

In this study, living ligation of bowel loops was firstly developed and applied for infection of rabbit gut associated lymphoid tissue in the sacculus rotundus and appendix by inoculating with HCMV solely or co-infecting with HCMV and HEV . PCR, western-blot, histopathology, enzyme immunohistochemistry and laser confocal immunofluorescence double labeling method were used to investigate the pathological changes of rabbit sacculus rotundus and appendix mucosal tissue caused by HCMY solely or HCMV and HEV synergistically infection, and the location of HCMV antigen. The results proved that HCMV DNA replication were detectable since 1.5 h post-injection by means of both genetic and protein technology. Meanwhile, the HCMV antigen was also detected in the lymphoid tissue of rabbit sacculus rotundus and appendix, via immunoenzyme labeling and immunofluorescence labeling.

Histopathological observation found that HCMV infected mucosa of SR and VA had damage, epithelium degeneration, congestion in lamina propria capillaries, lymphocytes evacuated, and/or necrosis. The morphology changes are more severe in HCMV + HEV co-infected tissue. It was characteristics by degenerated and exfoliated mucosal epithelium, hyperemia, edema and hemorrhage of lamina propria, which led to the exudation and drain or diffuse necrosis of lymphocytes in the deep layer, even syncytial giant cells formation in the mucosa lamina propria. These were all signs of inflammation, which indicated HCMV infection and propagation. Immunohistochemistry and immunofluorescence results revealed pp65 positive signals alongside the epithelium and Peyer’s patches of the sacculus rotundus and appendix, which suggested HCMV successfully infect these areas. There were also positive signals in the lamina propria, especially in the DI group, which indicated HCMV penetrated and reproduced in the rabbit intestinal tract; this was consistent with the RT-PCR signal detected in uninjected tissue.

Intestinal epithelium contains 15%–30% lymphocytes, which traversed from the lamina propria into the epithelium through the basilar membrane called IEL (intraepithelial lymphocyte) [33]. They are both thymus-dependent and thymus-independent. Their main functions are inhibition of intestinal mucosal hypersensitivity, recognition of foreign pathogens, and secretion of cytokine for anti-bacterial, anti-viral, and anti-canceration locally. In present study, the statistical data indicated that IELs increased largely in the mucosal epithelium and dome epithelium post-infection. The differences among the CT group, the SI group and the DI group were significant (*P* < 0.05). The increase was always accompanied by lymphocytes evacuated in deep lymphoid tissue, especially in the DI group, from histopathology observation. This indicated that lymphocytes may activate properly and migrate from deep inside tissue to the lumen during anti-viral process. The increase is also associated with activation of host immunity and this specific anti-viral immunity may be achieved through stimulation of local mucosal cellular immunity. The change of IGCs percentage was irregular. In the mucosal epithelium, IGCs secrete mucin as part of innate immunity so that IGCs percentage does not increase. In the unique dome epithelium, IGCs incorporated with M cells are specialized to sample local antigens. The significant increase of DA and SR tissue in different treatments suggested frequent antigen-presentation activities.

## Conclusions

The results above indicated that the rabbit sacculus rotundus and appendix were appropriate for HCMV infection and multiplication, and HCMV had a strong infectivity to these tissues as well. These results preliminarily proved that HCMV could reproduce and express its viral structural proteins in mucosal epithelial cell and lamina propria cells of the vivo rabbit ligated sacculus rotundus and vermiform appendix inoculated with HCMV. In the present study, the ligated intestine *in vivo* initiative proved HCMV could express its viral protein and cause serious inflammation damage in infected tissue of SI group and/or DI groups. Furthermore, this study revealed that the possibility of a rabbit model for HCMV intestinal infection is emerging, which provides a novel path for studying pathogenesis and prevention of HCMV infection.

We successfully established an *in vivo* rabbit intestinal loop model for human cytomegalovirus (HCMV) infection as demonstrated by pathological finding, the presence of viral DNA replication and viral protein expression with PCR, western blotting, immunohistochemistry and immunofluorescence assays. This study also showed that co-inoculation of HCMV and hepatitis E virus (HEV) to the animal model can promoted the pathogenicity of HCMV infection.
